# Knowledge, Attitudes, and Practices Regarding Dengue, Vector Control, and Vaccine Acceptance Among the Mexican Population

**DOI:** 10.7759/cureus.98389

**Published:** 2025-12-03

**Authors:** Fortino Solorzano-Santos, Miguel Betancourt-Cravioto, Gisel Escobedo, Jorge F Mendez-Galvan, Jose G Martinez-Nuñez, Abiel H Mascareñas de los Santos, Jesus F Gonzalez-Roldan, Sarbelio Moreno-Espinosa, Jose Ramos-Castañeda

**Affiliations:** 1 Research Laboratory for Infectious and Parasitic Diseases, “Federico Gomez” Children's Hospital, Mexico City, MEX; 2 School of Medicine, Anahuac Mayab University, Merida, MEX; 3 Medical Affairs, Takeda Mexico, Mexico City, MEX; 4 Infectious Diseases, “Federico Gomez” Children's Hospital, Mexico City, MEX; 5 International Technical Group on Arbovirosis, Pan American Health Organization, Monterrey, MEX; 6 Pediatric Infectious Diseases, "Dr. Jose E. Gonzalez" University Hospital, Universidad Autónoma de Nuevo León (UANL), Monterrey, MEX; 7 School of Medicine, Anahuac University, Mexico City, MEX; 8 Pediatric Infectious Diseases, Medica Sur Hospital, Mexico City, MEX; 9 Center for Research on Infectious Diseases, Instituto Nacional De Salud Pública, Cuernavaca, MEX

**Keywords:** dengue, knowledge, mexico, population survey, vaccine, vector control

## Abstract

Introduction

Dengue is a growing public health problem. Prevention strategies need to consider the population’s attitudes, practices, and knowledge about the disease, including its transmission and preventive measures. This study aims to analyze the results of the cross-sectional GEMKAP (Growth & Emerging Markets, Knowledge, Attitudes, and Practices) survey among the Mexican population and make recommendations for improvement.

Methods

A subset study of the GEMKAP survey was carried out, considering only Mexican participants. A total of 600 participants aged between 18 and 60 years were included. A web-based survey was administered from September to October 2022, with descriptive statistics applied for analysis.

Results

Six hundred people were evaluated; 55% were women, 39% were young adults, and 71% had at least a high school education. The scores obtained were: knowledge 48%, attitudes 68%, practices 42%, capability 55%, opportunity 61%, and motivation 58%. Knowledge regarding the vector varied; only 37% knew the four types of dengue virus, 18% were informed that co-infection with two or more types of the virus was possible, and 93% knew that dengue could be fatal. The majority (97%) reported implementing preventive measures for dengue. Confidence in vaccination was high (79%). The majority (80%) believed that the dengue vaccine should be available for everyone. Twenty-four percent would not get the vaccine if they had to pay for it, and 65% would get it to prevent dengue if a medical professional recommends it. The main concerns were the level of vaccine protection (46%) and safety (44%).

Conclusions

KAP studies provide valuable information obtained directly from the population affected by dengue. This data can help to improve dengue prevention and management strategies.

## Introduction

Dengue is a disease transmitted mainly by *Aedes aegypti* and *Aedes albopictus* and is a growing global public health problem [1,2]. Despite efforts to control the vector and the transmission of the virus, dengue is endemic approximately between latitude 30°N and 30°S worldwide, in what is known as the dengue belt, with recurrent outbreaks affecting millions of people each year [1,3-5]. The complexity of this disease lies not only in its biological mechanism of transmission but also in the interaction between social, economic, and environmental factors that impact the vulnerability of communities [4]. Therefore, effective strategies to prevent dengue must go beyond simple mosquito control and require modifying people’s attitudes, practices, and knowledge about the disease, including its transmission and preventive measures [1,3-5].

The scientific literature has extensively explored the relationship between knowledge, attitudes, and practices in preventing vector-borne diseases, including dengue [1,5]. PAHO issued guidelines for preventing and controlling dengue in the region, putting forward the Integrated Management Strategy for Dengue Prevention and Control (IMS-Dengue) [6]. This management model strengthens national programs aimed at reducing morbidity and mortality and the social and economic burden caused by dengue outbreaks and epidemics [6]. The model focuses on modifying individual and community behavior to diminish risk factors for transmission through coordinated measures inside and outside the health sector [6]. Previous studies have shown that a lack of information regarding the transmission of dengue and misconceptions surrounding the disease can make it difficult to implement effective preventive measures [1-3]. For example, it has been demonstrated that the exclusive use of larvicides as a vector control method is insufficient [7]. Furthermore, the perceived risk of contracting dengue, the public’s confidence in health authorities, and the availability of resources for prevention also influence the measures people take in their homes and communities [2-4].

In Mexico, an increasing trend in dengue incidence has been observed, with a notable growth in 2024. States such as Colima, Guerrero, and Tabasco have the highest rates of confirmed cases, with Colima ranking first at 140.11 cases per 100,000 inhabitants, followed by Tabasco (104.35) and Guerrero (101.83) [5,8]. In the latter, a significant increase in confirmed and estimated cases was documented, which is also reflected in the rising number of hospitalizations and severe cases reported across various states [9].

Furthermore, in Mexico, the transmission of dengue is highly variable across regions, from very low (<0.01) to incredibly high (>0.5) [10]. Notwithstanding, Mexico invests nearly half of its dengue budget in vector control strategies [11].

The Ministry of Health has confirmed that all four serotypes of the virus are currently circulating in the country, with a predominance of serotype 3 increasing the risk of severe and potentially fatal cases [9].

This increase in incidence highlights the need for preventive measures, including controlling the dengue-transmitting mosquito, raising public awareness about warning signs, and encouraging early medical attention.

This article explores the key roles of knowledge, attitudes, and practices of the Mexican population regarding dengue prevention. To this end, we conducted a sub-analysis of the data from the previously published GEMKAP (Growth & Emerging Markets, Knowledge, Attitudes, and Practices) study [12].

The aim of the study was to analyze the results of the GEMKAP survey among the Mexican population and to make recommendations for improvement.

## Materials and methods

The study GEMKAP was a multi-center (Argentina, Brazil, Colombia, Mexico, Indonesia, Malaysia, and Singapore), international, cross-sectional, and observational (quantitative electronic survey) study conducted in dengue-endemic countries [12]. 

The GEMKAP study used the Knowledge, Attitudes, and Practices (KAP) framework to assess the level of individuals’ knowledge living in endemic areas. It explored their education about dengue and its prevention, their attitudes toward the risk of contracting dengue, the effectiveness of vector control methods, and their attitudes and practices towards community-based dengue vector control, personal prevention, and prevention through vaccination [12-15]. The study applied the Capability, Opportunity, and Motivation for Behavior Change (COM-B) framework to identify the factors correlated with accepting the dengue vaccine(s) [12,15]. 

Only the responses of 600 Mexican participants from all areas of Mexico, 31 out of Mexico's 32 states, were considered in this subset study during the period September to October 2022 (see Appendices 1-5)**.** Details of the design and methodology can be found in the article by Shaffie et al. [12].

Participants

Inclusion Criteria

Potential respondents were individuals of both genders, ranging between 18 years old (legal age) and 60 years of age. The age limit was set at 60 to minimize differences in digital literacy among participants, thus reducing potential selection bias.

Exclusion Criteria

Additionally, individuals who had participated in other dengue-related surveys within the last three months were excluded, as were those who did not make decisions about their health or were not personally responsible for it.

A quota sampling procedure was applied, with strata based on gender, age, income, and regional distribution, to ensure the sample was representative of the country's gender, age, regional, and income distribution.

Most participants received an electronic invitation. To ensure adequate representation of older individuals and those living in remote areas without internet access in the total sample, respondents were recruited through local partner fieldwork agencies, inviting them to complete the survey at a partner center.

Instrument

A 35-item survey (binary answers (true/false), Likert scale, multiple-choice, and open-ended questions) previously published as material supplementary in Shaffie et al.'s study [12] was used. It took approximately 30 minutes to complete. The survey was designed in English and then translated into the native language of each country. The survey incorporated both frameworks: (i) KAP framework, aimed at identifying, understanding, and measuring misconceptions that could pose obstacles to activities or potential barriers to behavior change toward vaccination; (ii) COM-B framework, aimed at identifying factors that may influence individuals' capability, opportunity, and motivation, which can drive behavioral change for successful vaccine adoption [12]. 

Overall K, A, and P composite scores were also derived and standardized to a scale of 0-100%. For K, A, and P scores, 80-100% was considered a “high” score, 50-80% a “moderate” score, and 50% or below a “low” score. For attitude scores, a higher score indicated a more positive attitude [12]. 

The primary outcome sought was the respondents' willingness to receive the dengue vaccine. The study's secondary outcome was the general knowledge, attitudes, and practices regarding dengue infection and symptoms, dengue prevention methods, and dengue vaccines. For this report, four sections were considered: knowledge about dengue, general dengue prevention, prevention of diseases through vaccination, and prevention of dengue through vaccination. 

Statistical analysis

Sociodemographic variables, other baseline characteristics, and primary and secondary outcome variables were reported descriptively using simple frequencies and percentages for categorical variables and the mean for continuous variables. 

Ethics and data confidentiality

This subset study adhered to the scope detailed in the informed consent form signed by participants; therefore, it does not require Institutional Review Board (IRB) approval. Nonetheless, the primary project received approval from the Pearl Institutional Review Board (IN, USA; study number 22-VIST-101). Participants who completed the full survey received an incentive in the form of points that could be exchanged for a small prize.

## Results

Sociodemographic characteristics of the participants 

**Figure 1:**
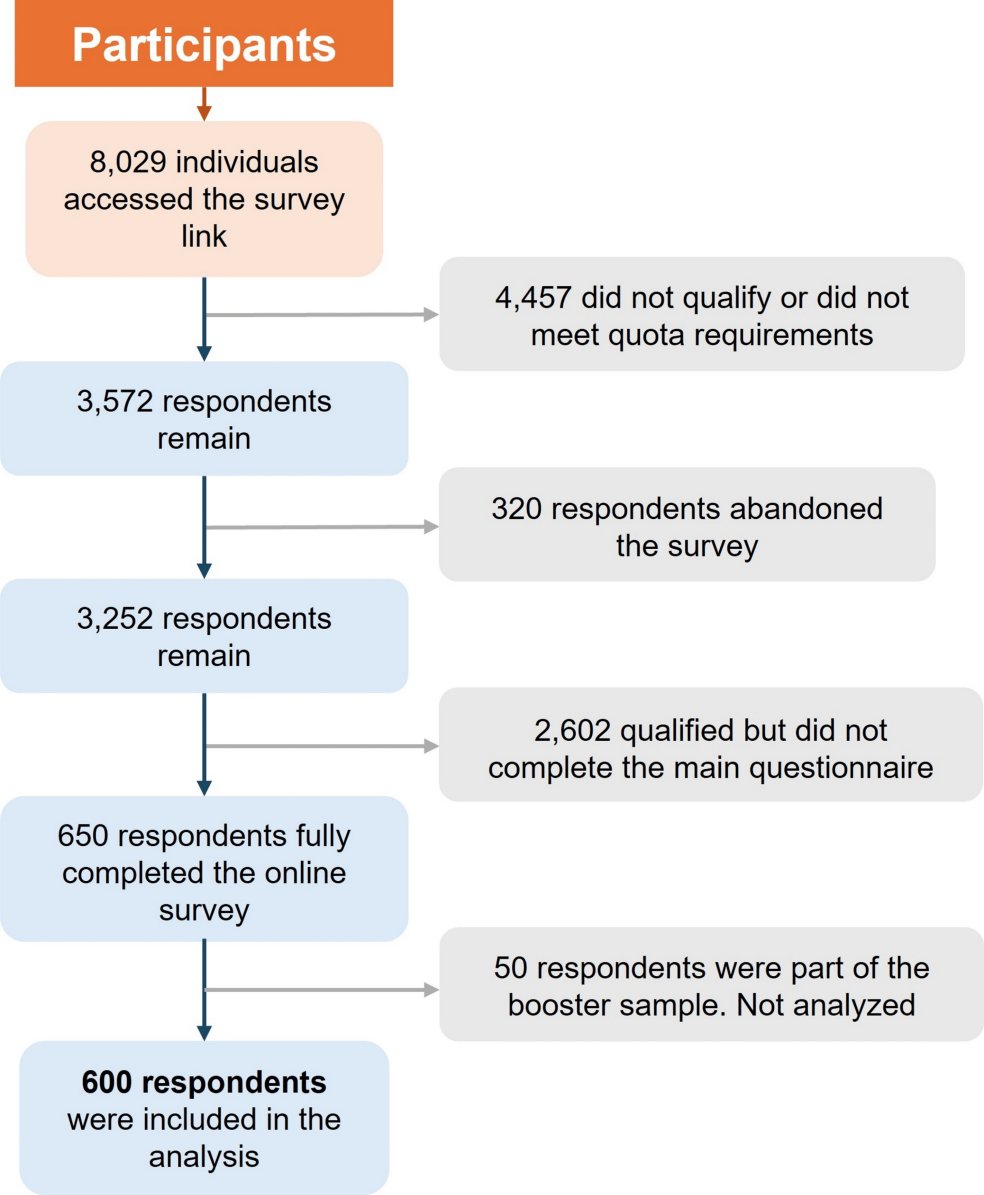
Participant flow diagram.

Out of 8,029 individuals who accessed the survey link from them 4,457 did not qualify or did not meet quota requirements; 320 respondents abandoned the survey during the screening test or main questionnaire; 2,602 qualified but did not complete the main questionnaire because the quota had already been filled; 600 respondents fully completed the online survey and were included in the analysis; and 50 respondents formed a booster sample but were excluded from the analysis (Figure 1). Among the 600 analyzed participants, 55% were women, 39% were young adults, 71% had at least a high school education, and 83% reported low (less than 9,000 Mexican pesos per month) to middle-income levels (from 9,000 to 39,000 Mexican pesos per month).

Regarding vaccination history, 94% reported having been vaccinated against COVID-19, and 76% had received the influenza vaccine. Questions about vaccination for dengue prevention and yellow fever prevention were not included. Additionally, 156 (26%) of respondents reported having previously contracted dengue.

Knowledge about dengue 

**Figure 2:**
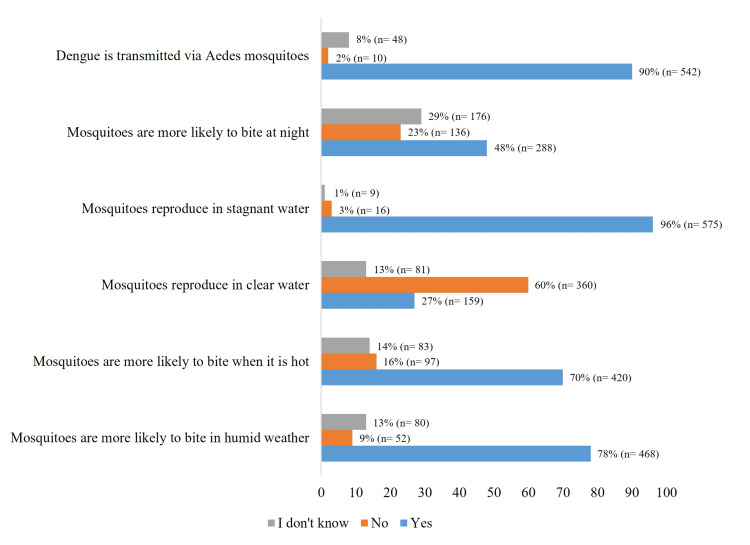
Dengue transmission statements. The data have been represented as percentage (n=number of participants)​.

Knowledge regarding the dengue vector was inconsistent. While most respondents were aware of the mosquito, fewer understood that it breeds in clear water, and less than 50% knew that the mosquito bites primarily during the afternoon and evening (Figure 2).

**Figure 3:**
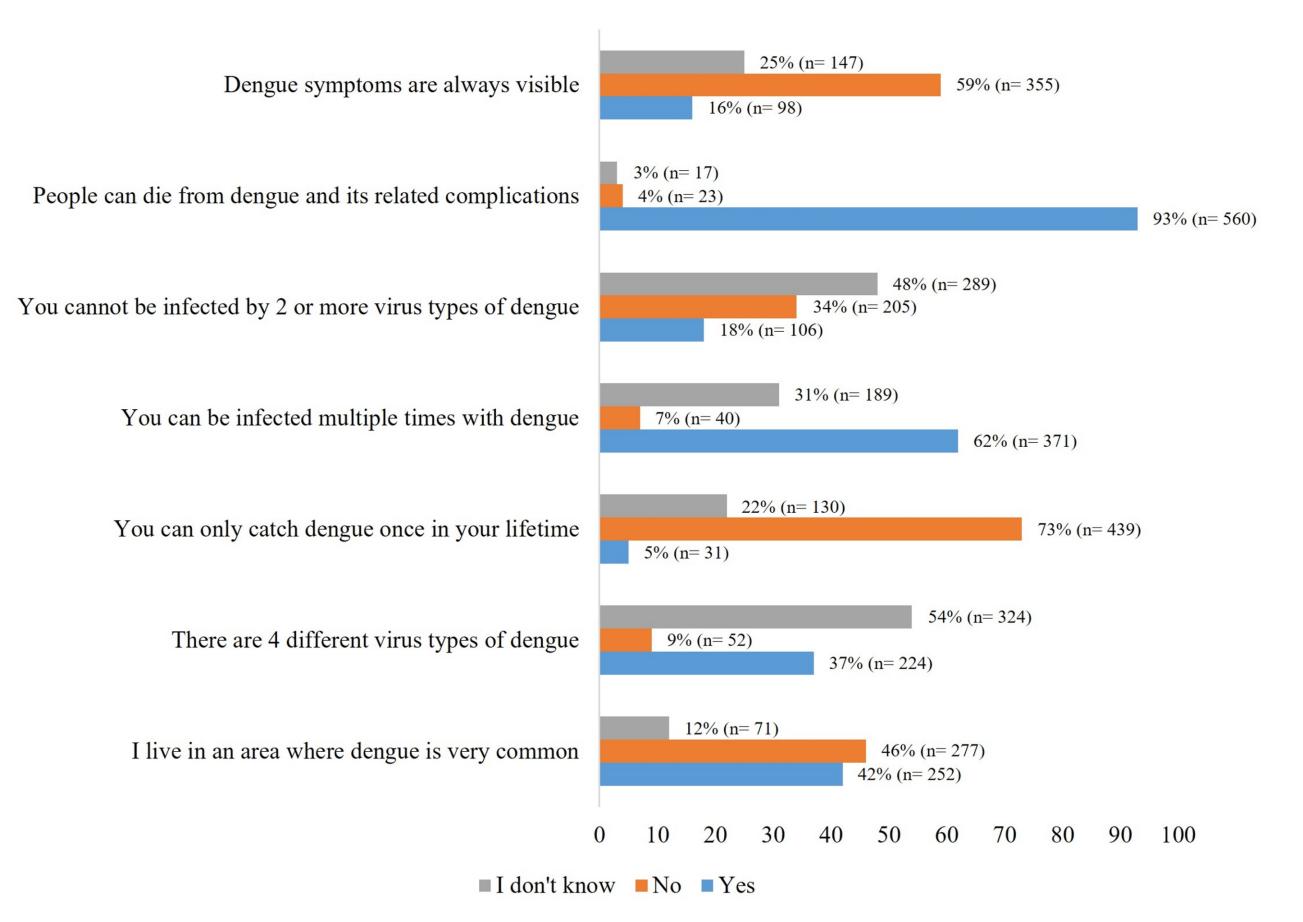
Dengue infection statements. The data have been represented as percentage (n=number of participants).

Regarding the dengue virus, only 37% knew that there are four types of dengue virus; 34% were aware that co-infection with two or more types of the virus was possible; 62% knew that a person could be infected with the virus multiple times; and 93% knew that dengue could be fatal (Figure 3). 

Most (71%) considered dengue a severe illness, and almost half of the respondents (44%) believed anyone could get infected. The population demonstrated a moderate level of knowledge about diagnostic methods. Sixty-nine percent correctly identified blood tests as the diagnostic method for dengue. 

In terms of clinical presentation, the three most recognized symptoms of dengue were fever (90%), headaches (79%), and generalized pain (76%). 

The most recognized consequences of dengue were potential medical visits (59%), potential hospitalization (57%), absenteeism (51%), and unexpected medical expenses (46%). The questionnaire did not ask whether death was a perceived consequence of dengue. However, awareness of secondary consequences was low. Only 11% recognized the impact on quality of life, 20% acknowledged a higher risk of reinfection, and 21% understood the potential severity of reinfection. 

General dengue prevention 

**Figure 4:**
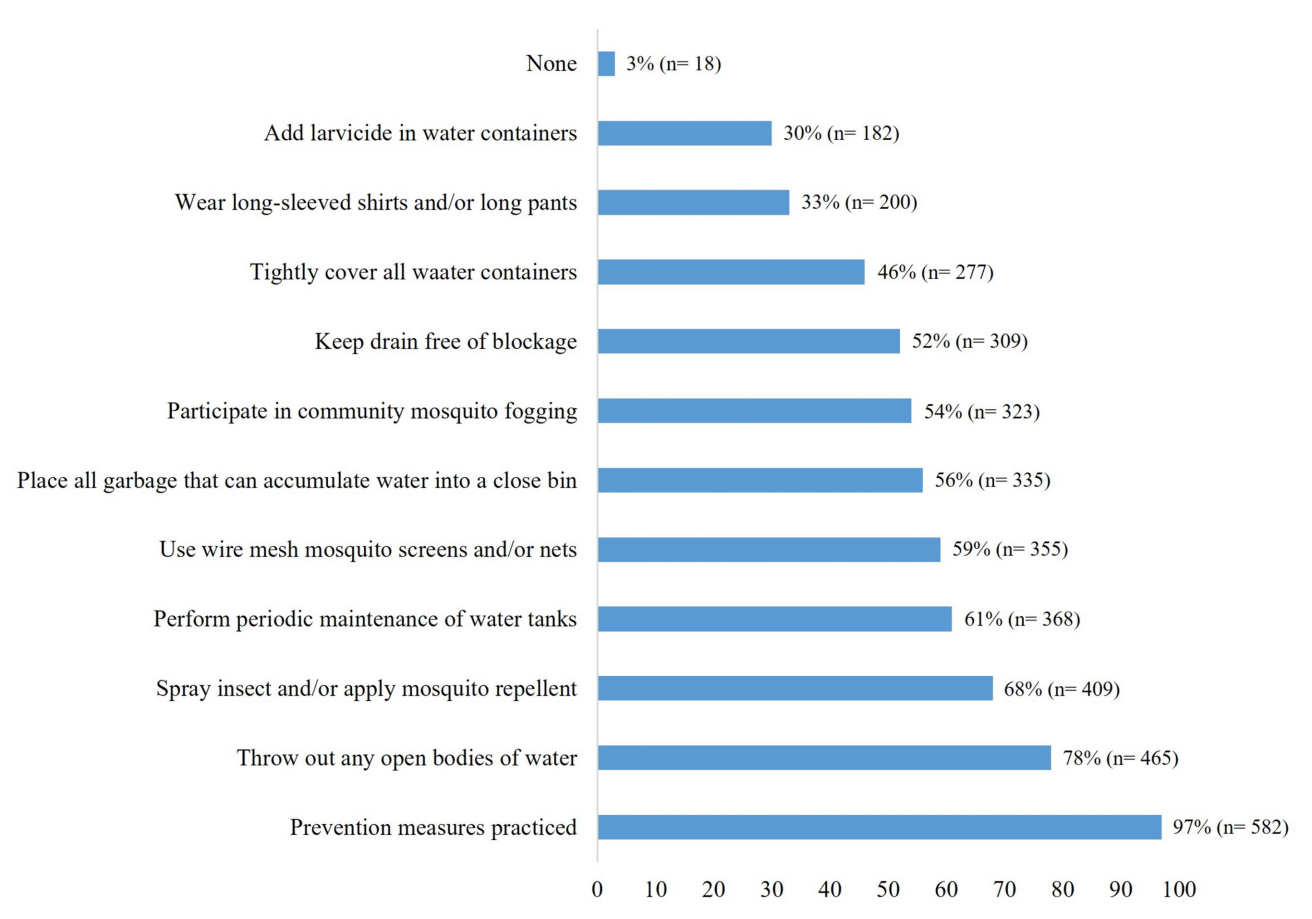
Frequency of prevention measures practices. The data have been represented as percentage (n=number of participants).

The majority (97%) of respondents reported practicing the 10 dengue preventive measures recommended by the Centers for Disease Control and Prevention (CDC) [16-20]. The most commonly implemented actions included eliminating standing water (78%), using insecticides or mosquito repellents (68%), regularly maintaining water tanks (61%), and installing mosquito screens or nets (59%) (Figure 4).

Prevention of diseases through vaccination 

**Figure 5:**
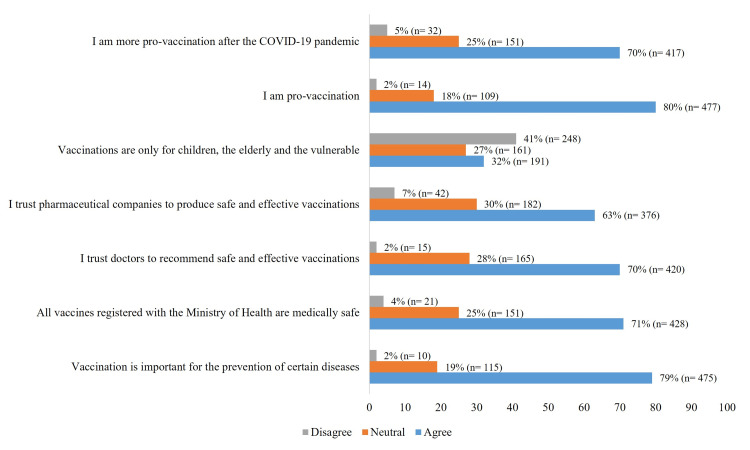
Impression of vaccines (general vaccines). The data have been represented as percentage (n=number of participants).

Overall, confidence in vaccination as a preventive measure against diseases was high (79%). Trust in the Ministry of Health and clinicians is similarly strong, at 70%. Confidence in the pharmaceutical industry as a producer of safe and effective vaccines is lower (63%); 32% of respondents believe that vaccination was primarily for children, older adults, and vulnerable populations (Figure 5).

Seventy-seven percent ensure their children are up to date with their vaccination schedules, 68% proactively ask their clinicians about vaccines, and, in turn, clinicians recommend vaccinations for various conditions (64%). 

The respondents' views on government vaccination measures were as follows: 61% agree that the government facilitates access to vaccines by providing convenient vaccination sites; 59% feel the government’s promotion of vaccination is low; and 51% believe that the government promotes the importance of vaccination. The decision to get vaccinated against influenza was primarily influenced by the vaccine being free (79%) and by awareness of the potential risks associated with not getting vaccinated (65%). In contrast, the factors that least influenced the decision to get vaccinated were material incentives (3%) and knowing someone who had an adverse reaction to the influenza vaccine (14%). 

The primary reasons for not getting the influenza vaccine were fear of side effects (28%), difficulty in scheduling an appointment (15%), and the cost of the vaccine (13%). 

Dengue prevention through vaccination 

**Figure 6:**
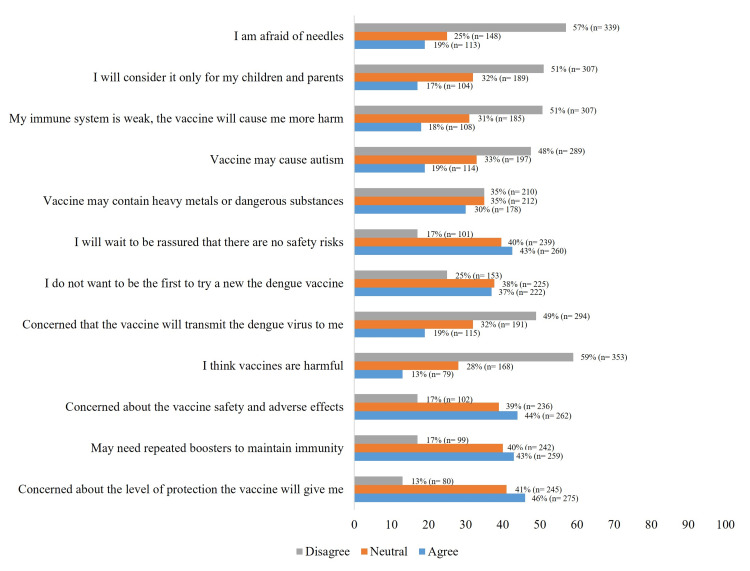
Impression of vaccines (dengue vaccine). The data have been represented as percentage (n=number of participants).

Key concerns about the dengue vaccine included its effectiveness (46%), safety (44%), the need for booster doses (43%), and the assurance of no adverse reactions (43%) (Figure 6). 

The majority (80%) believe that the vaccine should be available to the entire population, and 59% trust that health institutions and clinicians can manage both the administration of the vaccine and any adverse reactions to it. Nearly a quarter of the population (24%) would not get the vaccine if they had to pay for it. 

Sixty-five percent of respondents would get vaccinated to prevent dengue if their clinician recommended it. 

Most of the population (70%) believes that an education campaign should accompany the dengue vaccination program, and 32% also feel that it should be paired with a vector control program. 

The reasons the population gave for getting vaccinated against dengue were to prevent it (31%), to protect their health and well-being (25%), and to protect their family (18%). 

Conversely, the reasons reported for not getting vaccinated were adverse events (19%), lack of scientific evidence (7%), and lack of efficacy (5%). 

The preferred channels for obtaining health information were search engines (81%), social networks (59%), and television (41%). 

The survey question results for the Mexican population can be found in Appendices 1-5​​​​​​.

## Discussion

The global scores for each component of the KAP and COM-B frameworks in the GEMKAP study among the Mexican population were moderate (for interpretation, see reference [12]): knowledge 48%, attitudes 68%, practices 42%, capability 55%, opportunity 61%, and motivation 58%. These results underscore the need for population-level education on dengue and its prevention [12]. Considering the complexity of the elements involved in calculating these scores, analyzing the responses of the Mexican population could help identify areas for improvement.

The surveyed population had knowledge gaps regarding the transmission and symptoms of dengue, including certain misconceptions. This observation is consistent with the findings of Chuc et al., who reported that in Morelos, a region in central Mexico, individuals frequently confuse dengue with other infectious diseases, both in terms of its transmission and clinical presentation [4].

Vargas Navarro et al., in their systematic literature review on the general aspects of dengue and the challenges associated with technical and community participation in its eradication, identified the absence of self-directed community engagement in official prevention programs as the primary barrier to effective vector control [21]. They point out that in Mexico, vector control has historically followed a vertical preventive model, where technical government personnel are deployed to communities to inspect homes, yards, and surrounding areas to determine where larvicides (elimination) and insecticides (fogging) should be applied. Additionally, informational materials are disseminated through various media, bypassing active and self-directed participation from the communities [21]. The results of this study show that a low proportion of respondents cited community participation and self-management as useful tools for dengue control.

The Ministry of Health, through the National Center of Preventive Programs and Disease Control (CENAPRECE), published a guide based on the national by-law NOM-032-SSA2-2014, which recommends using community-based promotion as a key element for preventing and controlling dengue, and it outlines procedures to achieve these goals [22]. This guide is used by the health authorities, and they have mainly engaged communities during epidemics through large-scale "de-cluttering" campaigns, widespread distribution of printed materials, and radio and television campaigns to share information about the vector, its breeding grounds, and the disease itself. Public health efforts have traditionally focused on informing the population about the disease and its vector; however, existing public knowledge on the subject is rarely assessed [21]. As evidenced, community participation is not considered a significant and active element in the management of dengue. Therefore, it is necessary to determine the population's position on this issue (knowledge, attitudes, practices, and perception of preventive measures) to design better strategies for dengue control.

According to Vargas Navarro et al., only 33% of the reviewed literature reported on the lack of citizen participation in dengue control, citing minimal community involvement in vector elimination activities, a failure to assess public knowledge about disease prevention, and widespread reliance on routine larvicide and insecticide use as the main control strategy [21]. In contrast, the results of this study showed more participation of the community in the dengue control to prevent dengue, although, as mentioned previously, the fact that they carry them out does not mean that they trust them, as is the case with the application of larvicides. However, it is important to reinforce the community outreach, the meeting with social groups, and supervision and evaluation as mentioned in the guide to community participation in the prevention and control of vector-borne diseases [22].

The perception of dengue as a potentially severe disease, as observed in this study, contrasts with the findings of Menchaca-Armenta et al., who reported that in the general population of Hidalgo (a state with low dengue incidence), 93.1% of participants perceived the risk as low to moderate [5].

The Mongua-Rodríguez et al. study on knowledge, attitudes, and perceptions regarding the COVID-19 vaccine provides indirect information about the factors associated with vaccine hesitancy, including fear of adverse events and distrust of medical recommendations [23]. In the present study, the population showed greater confidence in vaccination, governmental health services, and the pharmaceutical industry.

Within this research, respondents showed moderate levels (50-80%) of satisfaction and confidence in the government’s ability to manage dengue. Likewise, they practiced a few prevention measures for dengue and did so infrequently. In general, studies conducted in Mexico support the following observation: Even when there is a high awareness of the severity of dengue and a high perceived risk of contracting it, the Mexican population tends to view disease control as the government's responsibility [24]. To the best of the authors' knowledge, although there has been a vaccine registry for several years, and it was available for purchase, no mass communication campaigns were carried out in the community, which could be related to this finding.

Although respondents reported greater trust in information provided by clinicians (92%) and only 25% trusted information provided by the government, it is worth noting that most of the population receives care from the public health system, and it is very likely that they were referring to institutional clinicians.

The findings of this study suggest that raising public awareness about the disease (recognition of its severity), as well as the effectiveness, safety, and benefits of the vaccine, could improve public health by increasing dengue protection, which could lead to higher vaccination rates and a reduced risk of severe dengue, hospitalizations, complications, quality-of-life impacts, and mortality. However, social media, the internet, and other media strongly influence the population, affecting the acceptance of a dengue vaccine [25].

At the same time, addressing misconceptions is essential, as many pose risks to public health - particularly two: the false sense of security that insecticide used alone interrupts dengue transmission and the mistaken belief that vaccination replaces integrated control measures when, in fact, it is just one additional tool to help prevent severe disease and deaths from dengue [26,27].

Strengths

This report is derived from the first multinational study conducted to understand knowledge, attitudes, and practices regarding dengue and its prevention. The study was designed to increase the generalizability and statistical power of the results. Online respondents underwent industry-standard quality controls, ensuring that the data collected came from reliable participants. 

Limitations

The study has five potential biases.

Sampling Bias

While the sample is geographically representative, it may not accurately reflect the distribution of disease transmission or the proportion of participants from each state. For instance, the classification between endemic and non-endemic areas was not precise, as they were not further categorized into endemic, hyperendemic, and non-endemic regions.

Selection Bias

A high proportion of participants were vaccinated against dengue. The study's objectives were deliberately excluded from the participant screening questions to reduce selection bias.

Recall Bias

Due to the self-administered nature of the survey, the high percentage of people vaccinated against dengue, and the fact that different areas have different epidemic curves for the disease, recall bias may have influenced responses.

Temporal Bias

The epidemiological context in which the survey was conducted (post-COVID-19 period) might have influenced respondents' willingness to vaccinate.

Availability Bias

The population may perceive more risk during or after an outbreak, as outbreaks occur at different times in different populations.

Recommendations 

In light of the results on public perceptions of dengue and its prevention and control strategies, the following recommendations are proposed.

Stronger Communication

Despite the longstanding implementation of structured information campaigns, their impact has been limited. Consequently, designing new messages and finding more effective information channels that directly address persistent misinformation and resonate with prevailing attitudes and perceptions is essential.

Policy Refinements

New health policies can formulate adjustments to dengue prevention and management strategies, addressing the identified gaps. 

Building Public Trust

Fostering greater public trust in health authorities is essential to improving the impact of health messages. At the same time, the concept of shared responsibility in developing and maintaining healthy environments should be actively promoted and strengthened. 

Vaccine Integration

Emphasizing the existence and potential usefulness of a safe and effective vaccine as an additional element in a multi-faceted strategy for preventing and controlling dengue.

## Conclusions

Considering that this study is exploratory, it shows that the population's knowledge of the vector and the virus was inconsistent. The population had limited knowledge about the mosquito's breeding sites and feeding habits, and a lack of awareness of the existence of four virus types and the possibility of co-infection. Although the majority of respondents claimed to follow the ten recommended vector control measures, actual adherence rates were below 70% for nine of them. Regarding attitudes towards vaccination in general, the population is pro-vaccination and trusts health authorities for its implementation. Regarding the vaccine to prevent dengue, there are concerns about its level of protection, the need for boosters, and its overall safety.

This study provides a comprehensive overview of the national population’s attitudes toward dengue and its prevention, offering areas of opportunity for community education and care. Dengue control requires addressing the problem from multiple perspectives: educational, community-based, governmental, medical, and pharmaceutical.

## Appendices

Aggregated results of the study survey for the Mexican population

Consider Q as a question.

Appendix 1 (dengue knowledge)

**Table 1:** Q1. Please select true or false for each statement.

STATEMENT	TRUE, %	FALSE, %	I DON’T KNOW, %
Dengue is transmitted to a person via Aedes mosquitoes	90	2	8
Aedes mosquitoes are more likely to bite in the evenings or at night	48	23	29
Mosquitoes reproduce OR multiply in stagnant water	96	3	2
Mosquitoes reproduce OR multiply in clear water	27	60	14
Mosquitoes are more likely to bite when the weather is hot	70	16	14
Mosquitoes are more likely to bite in humid weather. (Humidity refers to the amount of water vapor in the air. The higher the humidity, the higher the moisture in the air and the wetter it feels outside.)	78	9	13

**Table 2:** Q2. Please select true or false for each statement.

STATEMENT	TRUE, %	FALSE, %	I DON’T KNOW, %
I live in an area where dengue is very common	42	46	12
There are 4 different dengue virus types	37	9	54
You can only catch dengue once in your lifetime	5	73	22
1 or more dengue virus types may infect me at different points of time	62	7	32
2 or more dengue virus types cannot infect me at the same time	18	34	48
People can die from dengue and its related complications (e.g., fever, cough, etc.)	93	4	3
It is always possible to tell when someone has dengue by looking at them	16	59	25

**Table 3:** Q3. Have you or anyone else you know contracted dengue two times or more? Subset sample based on 'false' answer in question 'You can only catch dengue once in your lifetime' of Q2 section.

STATEMENT	YES, %
Yes, I have contracted dengue at least two times or more	14
Yes, I know someone who has contracted dengue at least two times or more	43
No, I do not know anyone who has contracted dengue at least two times or more	45

**Table 4:** Q4 and Q5. Please rate each statement on a scale from 0 to 10, with 0 being 'not severe at all' and 10 being 'very severe.' Likert scale, 10=very likely, 0 very unlikely.

Q4. How severe is dengue as a disease?					
STATEMENT	8-10 POINTS, %			4-7 POINTS, %	0-3 POINTS, %
How severe is dengue as a disease?	71			28	1
Q5. Thinking about yourself and your family, community and neighborhood, how likely is it that the following people will contract dengue?					
PEOPLE		8-10 POINTS, %	4-7 POINTS, %		0-3 POINTS, %
Yourself		25	50		25
Family		34	46		20
Older residents who live in the neighborhood, aged 65 years		42	42		17
People with chronic or long-term health conditions		42	41		17
Infants, children and adolescents of up to 18 years old		35	48		17
Adult residents, aged 18 - 65 years		38	48		14
Anyone		44	44		13

**Table 5:** Q6, Q7, and Q8. Please select all that apply. Non-exclusive answer (respondents can answer one or more options for that question, so it is not expected that the sum results in 100%).

Q6. How is dengue diagnosed?			
STATEMENT			YES, %
Blood test at the clinic or hospital			69
Signs/symptoms of the disease, e.g., sore throat, cough, fever. etc.			49
Previous medical and travel history			13
Self-testing blood kit performed at home			9
I do not know			9
Self-testing nasal swab kit performed at home			4
Medical scans			3
Q7. Which of the following can often be signs of dengue?			
SIGN		YES, %	
Fever		90	
Headache		79	
Body aches/joint and muscle pain		76	
Body chills		51	
Feeling very tired and sleepy		35	
Nausea and vomiting		30	
Swollen glands and lymph nodes		30	
Flushing (face and/or body becomes red)		29	
Pain behind the eyes		24	
Loss of appetite		22	
Body rashes		21	
Diarrhea		20	
Runny nose		20	
Bleeding from the nose or gums		18	
Difficulty breathing		17	
Abdominal/belly/stomach pain		15	
All of the above		3	
Hair loss (alopecia)		1	
Q8. If someone were to contract dengue, what might the consequences be?			
CONSEQUENCE	YES, %		
Potential clinic visits	59		
Potential hospitalization	57		
Absenteeism from school/work	51		
Additional unexpected treatment costs from stay at the hospital, e.g., blood tests	46		
Worsening of chronic and long-term health conditions	27		
Additional costs due to caregiver requirements (incl. absence from work)	23		
Increasing severity of dengue should it be contracted again	21		
Increased risk of contracting dengue again	20		
Poorer quality of life	11		
All of the above	9		

**Table 6:** Q9. How much do you agree with each of the following statements? Please rate each statement on a scale from 0 to 10, with 0 being strongly ‘disagree’ and 10 being ‘strongly agree.' Likert scale, 10=very likely, 0 very unlikely.

STATEMENT	8-10 POINTS, %	4-7 POINTS, %	0-3 POINTS, %
There is nothing we can do to treat dengue	10	21	69
There is nothing we can do to prevent dengue	9	20	72
The threat of dengue is or has been exaggerated by the media	8	26	67
The threat of dengue is or has been exaggerated by the government	11	26	63
The government is responding appropriately to combat dengue	26	48	26
The government is well prepared to combat dengue	21	45	35
We will all be completely powerless	13	36	50
We just have to accept it	13	31	56

Appendix 2 (dengue prevention)

**Table 7:** Q10. Based on your current knowledge about dengue, do you think the statements are true or false?

STATEMENT	TRUE, %	FALSE, %	I DON’T KNOW, %
There are specific medicines that can cure dengue	51	28	22
There is no vaccine yet that can prevent dengue	40	34	26
There is a vaccine that can prevent dengue, but it is not yet registered in my country	28	44	28

**Table 8:** Q11. Which of the activities are you currently practicing to prevent the transmission of dengue? Please select all that apply.

ACTIVITY	YES, %
Any mentioned (excluding none)	97
Throw out any open bodies of water found in plant containers, flowerpots, tires, etc.	78
Spray insect repellent and/or apply mosquito repellent patches	68
Perform periodic maintenance of water tanks	61
Use wire mesh mosquito screens and/or mosquito nets	59
Place all garbage that can accumulate water into a closed bin	56
Participate in community mosquito fogging	54
Keep drain free of blockage	52
Tightly cover all water containers	46
Wear long-sleeved shirts and/or long pants	33
Add larvicide in water containers to kill mosquito larvae	30
None of the above	3

**Table 9:** Q12. How often do you complete these dengue prevention activities? Please select the answer closest to what you do.

ACTIVITY	Spray insect repellent and/or apply mosquito repellent patches (N=409), %	Participate in community mosquito fogging (N=323), %	Wear long-sleeved shirts and/or long pants (N=200), %	Use wire mesh mosquito screens and/or mosquito nets (N=355), %	Throw out any open bodies of water found in plant containers, flowerpots, tires, etc. (465), %	Perform periodic maintenance of water tanks (N=368), %	Tightly cover all water containers (N=277), %	Keep drain free of blockage(N=309), %	Place all garbage that can accumulate water into a closed bin (N=335), %	Add larvicide in water containers to kill mosquito larvae (N=182), %
Always/nearly all the time	8	4	14	41	24	13	38	31	37	11
Every few hours/at least twice a day	7	2	7	4	6	3	7	6	6	3
Once a day	14	5	24	12	14	4	12	9	15	4
Once every other day	12	6	10	3	9	5	8	6	9	6
Once a week	15	11	10	5	14	15	6	13	9	15
Once every two weeks	12	15	8	6	8	11	7	9	5	9
Once a month	9	15	5	7	6	17	6	8	5	20
Less than once a month	9	9	7	5	5	12	5	7	4	8
As and when needed	8	19	11	13	8	14	8	7	6	14
None of the above	6	14	6	5	7	5	5	5	4	9

**Table 10:** Q13. How confident are you in completing these dengue prevention activities successfully? Please rate each statement on a scale from 0 to 10, with 0 being 'not confident at all' and 10 being 'very confident.' Likert scale, 10=very likely, 0 very unlikely.

POINTS	Spray insect repellent and/or apply mosquito repellent patches (N=383), %	Participate in community mosquito fogging (N=278), %	Wear long-sleeved shirts and/or long pants (N=189), %	Use wire mesh mosquito screens and/or mosquito nets (N=338), %	Throw out any open bodies of water found in plant containers, flowerpots, tires, etc. (N=434), %
8-10	61	61	63	78	79
4-7	35	33	35	20	18
0-3	4	6	2	2	3

**Table 11:** Q14, Q15, and Q16. Please rate each statement on a scale from 0 to 10, with 0 being 'not confident at all' and 10 being 'very confident.' Likert scale, 10=very likely, 0 very unlikely.

Q14. How effective do you think each of the dengue prevention methods is for your personal health?			
STATEMENT	8-10 POINTS %	4-7 POINTS %	0-3 POINTS%
Use wire mesh screens, mosquito nets, and/or mosquito coils	62	35	3
Drain water from pots and cover all water containers	73	24	3
Community mosquito fogging	72	26	2
Spraying mosquito repellent and using larvicide to kill mosquito larvae	64	32	4
Wolbachia program (WIAM)	51	43	7
Dengue vaccination	65	30	5
Q15. How safe do you think each of the following dengue prevention methods are for your personal health?			
STATEMENT	8-10 POINTS %	4-7 POINTS %	0-3 POINTS%
Use wire mesh screens, mosquito nets and/or mosquito coils	67	31	2
Drain water from pots and cover all water containers	75	23	2
Community mosquito fogging	70	27	3
Spraying mosquito repellent and using larvicide to kill mosquito larvae	67	29	4
Wolbachia program (WIAM)	56	38	6
Dengue vaccination	67	28	5
Q16. How likely are you, your community/neighborhood/local council leader(s), or your government leader(s) (e.g., governors, mayors, councilors, etc.) to realize the following dengue prevention activities in the next 6 months?			
STATEMENT	8-10 POINTS, %	4-7 POINTS, %	0-3 POINTS, %
Use wire mesh screens, mosquito nets, and/or mosquito coils	51	39	11
Drain water from pots and cover all water containers	57	35	8
Community mosquito fogging	51	38	11
Spraying mosquito repellent and using larvicide to kill mosquito larvae	50	38	12
Wolbachia program (WIAM)	31	46	23
Dengue vaccination	45	39	16

Appendix 3 (dengue prevention with vaccine)

**Table 12:** Q17 and Q18. Please rate each statement on a scale from 0 to 10, with 0 beings 'strongly disagree' and 10 beings 'strongly agree.' Likert scale, 10=very likely, 0 very unlikely.

Q17. How much do you agree with the statements below on vaccines as a preventative activity in general?			
STATEMENT	8-10 POINTS %	4-7 POINTS %	0-3 POINTS%
Vaccination is important for the prevention of certain diseases	79	19	2
All vaccines registered with the Ministry of Health are medically safe	71	25	4
I trust clinicians to recommend safe and effective vaccinations	70	28	3
I trust pharmaceutical companies to produce safe and effective vaccinations	63	30	7
Vaccinations are only for children, the elderly, and the vulnerable	32	27	41
I am pro-vaccination	80	18	2
I am more pro-vaccination after the COVID-19 pandemic	70	25	5
Q18. Based on your past vaccination experience (in general), how much do you agree with the statements below?			
STATEMENT	8-10 POINTS, %	4-7 POINTS, %	0-3 POINTS, %
My clinician recommends my family and me vaccines for several health conditions as appropriate (N=599)	64	29	6
I make sure that my children's vaccination schedule is followed (N=402)	77	20	3
I proactively ask my family's or children's doctor about vaccinations (N=402)	68	26	6
I receive reminders from clinicians/the government about my upcoming vaccinations (N=599)	50	30	19
The government has broadcasted education campaigns for people to get vaccinated (N=599)	59	32	9
The government has made it easy for people to get vaccinated by offering vaccines at convenient locations (N=599)	61	32	7
My community/government leader(s) (e.g., governors, mayors, councilors, etc.) promote the importance of vaccines (N=599)	51	36	13
My favorite influencer(s) (e.g., local and international celebrities, etc.) promote(s) the importance of vaccines (N=599)	39	38	23
It is easy to schedule a vaccination appointment (N=599)	55	34	11

**Table 13:** Q19 and Q20. Please select all the statements that played a part in your decision to take the influenza vaccine. Non-exclusive answer (respondents can answer one or more options for that question, so it is not expected that the sum results in 100%). *Based on 'Those NOT vaccinated for influenza'; NA: Not applicable.

Q19. Based on your past vaccination experience		
STATEMENTS	YES, %	
The vaccination was free, discounted, or the cost was claimed back from the government or insurance company	79	
I was aware of the consequences if I did not vaccinate	65	
It was recommended by my clinician	49	
The vaccine's benefits outweigh the side effects	48	
It was recommended by the government	46	
There was sufficient scientific evidence of the vaccine's safety and effectiveness	45	
I was not afraid of the vaccine's side effects	42	
I wanted to protect my friends and family from contracting influenza	41	
It is easy to get a vaccination appointment	32	
It contributed to herd immunity (where a large part of the population is immune to influenza, reducing the spread of the disease in the general population)	28	
I felt that I was at high risk of contracting influenza	25	
My friends and family encouraged me to receive the vaccine	19	
I heard about a friend/family who had developed severe symptoms from influenza	14	
I was given the vaccine during a routine visit to a clinician	14	
I received an incentive (cash, points, or a present) to take the vaccine	3	
Q20. Please select all the statements that played a part in your decision not to receive the influenza vaccine (n=145*)		
STATEMENT		YES, %
I was afraid of the side effects that I might experience from the vaccine		28
It is not easy to get a vaccination appointment		15
The vaccination was not free/discounted/claimed from the government or insurance company		13
The vaccination was not affordable		13
There was not sufficient scientific evidence on the vaccine's safety and how well it protects against influenza		12
I am afraid of needles		12
I think that the vaccine is not safe		10
I did not think that I was at risk of contracting influenza		10
It was not recommended by my clinician		10
I heard about a friend/family who had developed severe side effects from the influenza vaccine		8
It was not recommended by the government		8
I experienced a side effect(s) from the vaccine, and I am reluctant to receive further vaccination		7
The vaccines' side effects outweighed their benefits		7
There would be no consequences if I did not get vaccinated		6
My friends and family discouraged me from taking the vaccine		4
I had been previously advised against receiving a vaccination		3
Even if I was sick with influenza, I do not think I would infect others with influenza		3
Only for certain ages, e.g., older adults, pregnant women		2
Not available, not enough vaccines, shortage		2
It does not contribute to herd immunity (where a large part of the population is immune to influenza, reducing the spread of the disease in the general population)		1
Forgot to vaccinate		1
No vaccination campaigns, not well-known		1
I am vaccinated; I will get vaccinated		1
I don't know, I had no information, and it was not clear		1
There was no incentive (cash, points, or a gift) to take the vaccine		NA

**Table 14:** Q21, Q22, Q23, and Q24. Please rate each statement on a scale from 0 to 10, with 0 beings 'strongly disagree' and 10 beings 'strongly agree.' Likert scale, 10=very likely, 0 very unlikely.

Q21. Assuming a vaccine is developed for dengue prevention and approved by global and local health authorities, how much do you agree with the statements below?							
STATEMENT	8-10 POINTS, %			4-7 POINTS, %			0-3 POINTS, %
I am concerned about the level of protection the vaccine will give me	46			41			13
I am concerned that I may need repeated booster vaccines to maintain immunity	43			40			17
I am concerned about the vaccine’s safety and adverse effects	44			39			17
I think vaccines are harmful	13			28			59
I am concerned that the vaccine will transmit the dengue virus to me	19			32			49
I do not want to be the first to try a new dengue vaccine	37			38			26
I will wait to be reassured that there are no safety risks	43			40			17
I am concerned that the vaccine may contain heavy metals or dangerous substances	30			35			35
I am concerned that the vaccine may cause autism	19			33			48
My immune system is weak, and I believe that taking the vaccine will do me more harm	18			31			51
I will consider it only for my children or parents	17			32			51
I am afraid of needles	19			25			57
I don't believe in vaccines	13			22			66
Q22. How much do you agree with the statements below regarding a hypothetical dengue vaccine?							
STATEMENT	8-10 POINTS, %	4-7 POINTS, %				0-3 POINTS, %	
I believe that the vaccine should be made accessible to the public, including myself	80	17				3	
I trust the healthcare system and professionals in my country to administer the vaccine and manage its side effects	59	33				8	
If I have to pay for the vaccination, I will not get it	24	41				35	
I will be more willing to get vaccinated if there are incentives (cash, points, or a gift)	23	32				45	
It depends on how severe the dengue epidemic is/how common dengue is when I am offered the vaccine	30	42				29	
If the risk of contracting dengue is low, I may not get the dengue vaccine	25	35				41	
I think dengue vaccination is more important than other optional vaccines (e.g., influenza)	19	49				32	
I am not convinced that it will be effective; look at the influenza/COVID-19 vaccine	18	39				43	
Q23. Assuming a vaccine is developed for dengue prevention and approved by global and local health authorities, how willing are you to consider getting vaccinated or recommend vaccination against dengue?							
STATEMENT	8-10 POINTS, %				4-7 POINTS, %		0-3 POINTS, %
Myself	64				31		5
My spouse (if applicable)	55				27		5
My children (if applicable)	55				24		6
My parents (if applicable)	62				27		6
My friends	55				38		8
Q24. Are you willing to consider a dengue vaccine if it is recommended by your clinician?							
STATEMENT	8-10 POINTS, %		4-7 POINTS, %				0-3 POINTS, %
I am willing to consider a dengue vaccine if it is recommended by my physician	65		32				4

Appendix 4 (dengue vaccination + vector control)

**Table 15:** Q25, Q26, Q27, and Q28. Non-exclusive answer (respondents can answer one or more options for that question, so it is not expected that the sum results in 100%).

Q25. Do you think that a dengue vaccination program should be rolled out with other programs, or as a standalone program? Other programs may include dengue vaccine education, vector control (any method to limit or eliminate mosquitoes that transmit dengue), vector control education, etc.		
STATEMENT		YES, %
I believe that a vaccination program should be rolled out with an education program		38
I believe that a vaccination program should be rolled out with both an education program and a vector control program		32
I believe that a vaccination program should be rolled out with a vector control program (any method to limit or eradicate mosquitoes that transmit dengue)		17
I believe that a vaccination program should be a standalone program		10
I believe that a vector control program alone is sufficient		2
I don't believe a vaccination program is necessary		2
Q26. Please share any other reason why you would be willing to get a dengue vaccine.		
REASONS	%	
Scientific benefits	66	
Avoid sickness/prevent dengue/protect against dengue	31	
Health and wellbeing	40	
Take care of myself/to protect my health and wellbeing	25	
Protect my family/children	18	
Personal belief	20	
Circumstances	6	
Others	5	
Q27. Please share any other reason why you would not be willing to get a dengue vaccine.		
REASON	%	
Scientific reasons	38	
Side effects/adverse effects/complications/consequences	19	
Lack of safety information/studies/no scientific evidence/no knowledge/lack of reliable third-party information	7	
Not effective/lack of quality/ineffective/not sure of effectiveness	5	
Personal beliefs	16	
I will get vaccinated if research is supported/scientifically proven, only in dengue outbreak areas, only if the vaccine is covered/claimable/reimbursed, or only if it is free of cost	13	
Circumstances	10	
I will get vaccinated	7	
Q28. Please select which dengue prevention activities the government should allocate more resources to (e.g., funding, subsidizing, distribution, manpower) in the future.		
DENGUE PREVENTION ACTIVITY	%	
Community mosquito fogging	77	
Dengue vaccination	70	
Spraying mosquito repellent and using larvicide to kill mosquito larvae, incl. fungicide	48	
A natural bacteria called Wolbachia reduces the abilities of the Aedes mosquitoes to transmit dengue or hybrid mosquito	44	
Use wire mesh screens, mosquito nets, or mosquito coils	40	
Drain water from pots, drains, and other sites and cover all water containers	39	

Appendix 5 (education)

**Table 16:** Q29, Q30, and Q31. Likert scale, 10=very likely, 0 very unlikely.

Q29. Do you agree with the statements below on the impact of religion on your daily decision-making process? Please rate each statement on a scale from 0 to 10, with 0 beings 'strongly disagree' and 10 beings 'strongly agree.' Religion and spirituality may impact decisions related to, but not limited to, diets, medicines with animal origins (e.g., halal vaccines, sterile insect treatments, blood transfusions, etc.), and a preferred gender of clinicians or other healthcare professionals.						
STATEMENT	8-10 POINTS, %			4-7 POINTS, %	0-3 POINTS, %	
My religious beliefs guide my health decisions	11			20	69	
I live my life according to my religious beliefs	16			28	56	
My religion prohibits me from getting vaccinated	7			10	82	
Q30. Do you agree with the following statements relating to your community/government leader(s) (e.g., governors, mayors, councilors etc.)? Please rate each statement on a scale from 0 to 10, with 0 beings 'strongly disagree' and 10 beings 'strongly agree.' Community/government leader(s) can include village chiefs, community health leaders, residential community leaders, etc.						
STATEMENT		8-10 POINTS, %	4-7 POINTS, %			0-3 POINTS, %
The opinion of my community/government leader(s) is important to me		23	43			34
My community organizes events promoting health		30	44			26
I actively participate in community events specific to promoting good/improved health and wellbeing		24	46			31
Q31. Do you agree with the following statements relating to your influencer(s)? Please rate each statement on a scale from 0 to 10, with 0 beings 'strongly disagree' and 10 beings 'strongly agree.' Influences can include social media influencers, local and international celebrities, etc.						
STATEMENT		8-10 POINTS, %	4-7 POINTS; %			0-3 POINTS, %
The opinions of my influencer(s) are important to me		18	39			43
My influencer(s) organizes events promoting good/improved health and wellbeing		22	41			37
I actively participate in health-related events hosted by my influencer(s)		19	38			43

**Table 17:** Q32, Q33, Q34, and Q35. Non-exclusive answer (respondents can answer one or more options for that question, so it is not expected that the sum results in 100%).

Q32. Which of the following channels do you most commonly use when actively looking for health-related information, including information about vaccines? Please select all that apply.			
CHANNEL		%	
Search engines (e.g., Google, Yahoo!)		81	
Social media (e.g., Facebook, Twitter, TikTok, Instagram, YouTube)		59	
Television		41	
Websites specializing in health-related information (e.g., WebMD, Mayo Clinic, Hello Health)		36	
Government or health agency websites or portals (e.g., Ministry of Health, Ministerio de Salud, Ministério da Saúde, Kementerian Kesihatan)		36	
Product and brand websites		26	
Messaging platforms (e.g., WhatsApp, Telegram, Facebook Messenger)		25	
Scientific journals (e.g., PubMed, New England Journal of Medicine)		23	
Radio		19	
Newspapers, magazines, media prints, journalists (e.g., Iker Jiménez)		18	
Consumer reviews		17	
Online forums, 'Question & Answer' services (e.g., Quora, Reddit)		14	
Product and brand blogs/video blogs		12	
Patient advocacy websites		6	
Q33. Out of the following channels that you use, please select the top 3 channels that you most commonly use when looking for health-related information.			
CHANNELS	%		
Search engines (e.g., Google, Yahoo!)	74		
Social media (e.g., Facebook, Twitter, TikTok, Instagram, YouTube)	50		
Television	26		
Websites specializing in health-related information (e.g., WebMD, Mayo Clinic, Hello Health)	26		
Government or health agency websites or portals (e.g., Ministry of Health, Ministerio de Salud, Ministério da Saúde, Kementerian Kesihatan)	22		
Messaging platforms (e.g., WhatsApp, Telegram, Facebook Messenger)	14		
Scientific journals (e.g., PubMed, New England Journal of Medicine)	12		
Product and brand websites	9		
Newspapers, magazines	6		
Consumer reviews	6		
Radio	6		
Online forums, 'Question & Answer' services (e.g., Quora, Reddit)	4		
Product and brand blogs/video blogs	3		
Patient advocacy websites	3		
Others	1		
Q34. Which of the following types of people/organizations do you trust to receive health-related information from?			
PEOPLE/ORGANIZATION			%
Clinicians			92
Nurses/Paramedics			46
Scientific organizations, incl. biologists			36
Pharmacists			26
Government			25
Non-profit organizations (e.g., World Health Organization)			24
Pharmaceutical companies			18
Community leaders (e.g., village chiefs, community health leaders, residential community leaders, etc.)			5
Influencers (e.g., local and international celebrities, etc.), incl. Iker Jimenez			3
Religious leaders			2
Family, friends and/or colleagues			1
Others			1
Q35. Out of the types of people/organizations that you trust, please select the top 3 sources that you trust the most when looking for health-related information.			
PEOPLE/ORGANIZATION			%
Clinicians			91
Nurses/Paramedics			39
Scientific organizations			28
Pharmacists			20
Non-profit organizations (e.g., World Health Organization)			19
Government			18
Pharmaceutical companies			11
Family, friends and/or colleagues			11
Community leaders (e.g., village chiefs, community health leaders, residential community leaders, etc.)			2
Religious leaders			1
Influencers (e.g., local and international celebrities, etc.)			1
Others			1
